# Inflammation-Associated Mechanisms of Blood–Brain Barrier Disruption and Depression Pathogenesis in People with and Without HIV

**DOI:** 10.3390/cells15050399

**Published:** 2026-02-25

**Authors:** Caitlin Hills, Cristian A. Hernandez, Vilma Gabbay, Joan W. Berman

**Affiliations:** 1 Department of Pathology, Albert Einstein College of Medicine, Bronx, NY 10461, USA; caitlin.hills@einsteinmed.edu (C.H.); cristian.hernandez@einsteinmed.edu (C.A.H.); 2 Nathan Kline Institute for Psychiatric Research, Orangeburg, NY 10962, USA; vxg595@med.miami.edu; 3 Department of Psychiatry, University of Miami School of Medicine, Miami, FL 33136, USA

**Keywords:** depression, blood–brain barrier, peripheral mediators, neuroinflammation, HIV, neuropsychiatric disorders, cellular mechanisms, neuroimaging

## Abstract

Depression is the most common neuropsychiatric comorbidity in people with HIV (PWH), with a prevalence of 30–50%, nearly twice that of the general population. Depression is a major cause of disease burden worldwide associated with increased morbidity and mortality in both people with and without HIV. Converging lines of evidence indicate that chronic peripheral inflammation and neuroinflammation, blood–brain barrier (BBB) disruption, and neurocircuit-level changes interact to mediate depression pathogenesis, and that these processes may be especially relevant in PWH. HIV-associated chronic inflammation, which persists despite viral suppression with antiretroviral therapy, may contribute to depression pathogenesis in this population. BBB permeability has been hypothesized to serve as a key mediator for the interaction of peripheral inflammation with the central nervous system in depression pathogenesis. In this review, we will describe the structure and function of the BBB and how peripheral inflammation interacts with cells of the BBB and the mechanisms that lead to increased BBB permeability. We will discuss current research addressing how peripheral inflammation and BBB disruption contribute to depression pathogenesis in people with and without HIV. We will review current techniques for studying BBB permeability in in vitro, animal, and clinical models and outline future directions for ongoing research.

## 1. Introduction

HIV remains a major public health problem, with over 40 million people living with HIV as of 2024 [1]. Despite the efficacy of antiretroviral therapy (ART) in controlling HIV infection and greatly prolonging lifespan, people with HIV (PWH) still develop HIV-associated neurocognitive comorbidities. Depression is the most common neuropsychiatric disorder in PWH, estimated to affect 30–50% of PWH [2,3,4,5,6,7]. This is almost twice the prevalence in the general population [7,8,9]. Depression in PWH is associated with increased progression of HIV disease, viral transmission, and mortality and hinders HIV eradication strategies [4,10,11,12]. HIV-associated chronic inflammation, which persists despite viral suppression with ART [10,13,14], may contribute to depression pathogenesis in this population. Blood–brain barrier (BBB) permeability has been hypothesized to serve as a key mediator for the interaction of peripheral inflammation with the central nervous system (CNS) in HIV-associated depression pathogenesis.

The BBB is a key interface between the peripheral circulation and the CNS as it regulates the transit of mediators and peripheral blood cells into the CNS [15,16]. The BBB is formed primarily by the interaction of brain microvascular endothelial cells (BMVECs), astrocytes, mural cells including pericytes, and other non-cellular components. Tight junction proteins including claudin-5, occludin, and zona occludens (ZO)-1 form junctions between BMVECs and maintain BBB integrity. Decreased tight junction proteins, and subsequent BBB disruption, have been implicated in depression pathogenesis in mice [17,18]. There is evidence that inflammatory mediators that are increased in both HIV infection and depression, including tumor necrosis factor-alpha (TNFα), interleukin-1beta (IL-1β), and interleukin-6 (IL-6), disrupt the BBB through various mechanisms. Transmigration of HIV-infected immune cells into the CNS may also contribute to this disruption, but these mechanisms are unclear.

In this review, we will describe the normal structure and function of the BBB, how peripheral inflammation interacts with cells of the BBB, and the mechanisms that lead to increased BBB permeability. We will also examine current research on how peripheral inflammation and BBB disruption contribute to depression pathogenesis in people with and without HIV. Lastly, we will review current techniques for studying BBB permeability in in vitro, animal, and clinical models and outline future directions for ongoing research. This is the first review, to our knowledge, to examine the interrelationship among inflammation, BBB disruption, and depression in the context of HIV.

## 2. Structure and Function of BBB

**Figure 1:**
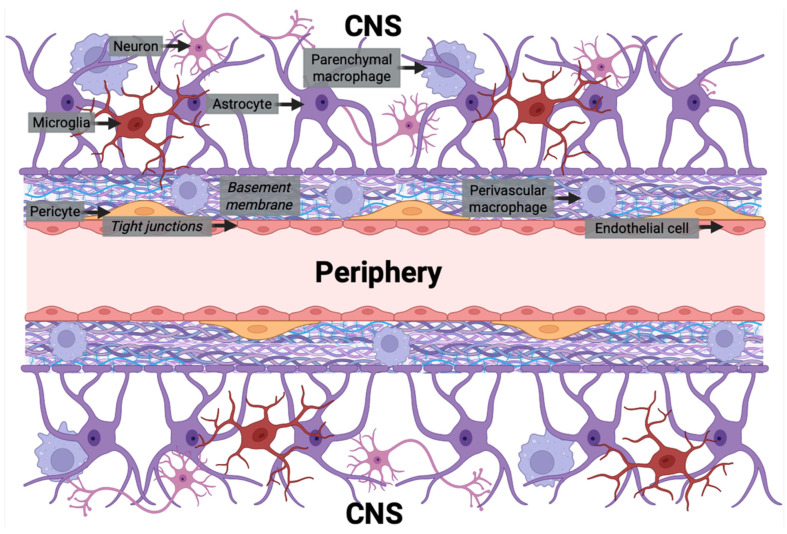
A diagram of the blood–brain barrier and other cells of the neurovascular unit. The BBB regulates the exchange of ions, nutrients, cells, and signaling molecules between the peripheral circulation and the brain to maintain central nervous system (CNS) homeostasis. Core structural components include brain microvascular endothelial cells (BMVECs), which are connected by tight junction proteins including claudin-5, occludin, and ZO-1; pericytes embedded within the basement membrane; and astrocytes whose endfeet envelop the vasculature. Other neurovascular unit cells, including macrophages, microglia, and neurons, are not structural elements of the barrier but interact closely with BBB cells to regulate permeability, vascular tone, and barrier maintenance. Perivascular macrophages are found in the perivascular spaces, while parenchymal macrophages are in the brain parenchyma. Together, these coordinate to preserve BBB integrity and CNS function. Figure created with Biorender by authors.

The cerebral capillaries that form the BBB regulate the influx and efflux of ions, nutrients, and signaling factors between the peripheral circulation and brain parenchyma to maintain CNS homeostasis and neuronal function. The BBB consists of various cellular and non-cellular components, all of which contribute to its physiological structure and function. Cellular constituents of the BBB include BMVEC, pericytes, and astrocytes. However, other cells, including macrophages, microglia, and neurons, which are not structural components of the BBB, interact closely with its cells to regulate barrier integrity and function. Collectively, these cells form the neurovascular unit and coordinate to preserve CNS homeostasis. The properties of the BBB are also discussed extensively in other reviews [15,16,19,20]. These cellular and non-cellular elements of the BBB are described below and illustrated in Figure 1.

### 2.1. Endothelial Cells

Brain microvascular endothelial cells (BMVECs) are a major component of the BBB. BMVECs are specialized cells that, unlike endothelial cells that line the peripheral blood vessels, do not have fenestrations. This lack of fenestrations decreases transcellular transport of ions, molecules, and cells [20,21,22]. BMVECs also express specific transporters and efflux pumps, such as glucose transporter 1 (GLUT-1) and P-glycoprotein (P-gp), that transfer the necessary nutrients into and waste products/toxins out of the CNS [19,20]. Additionally, compared to peripheral endothelium, BMVECs have lower expression of leukocyte adhesion molecules including selectins and immunoglobulin family adhesion molecules that limits transmigration of immune cells into the CNS [19,20].

**Figure 2:**
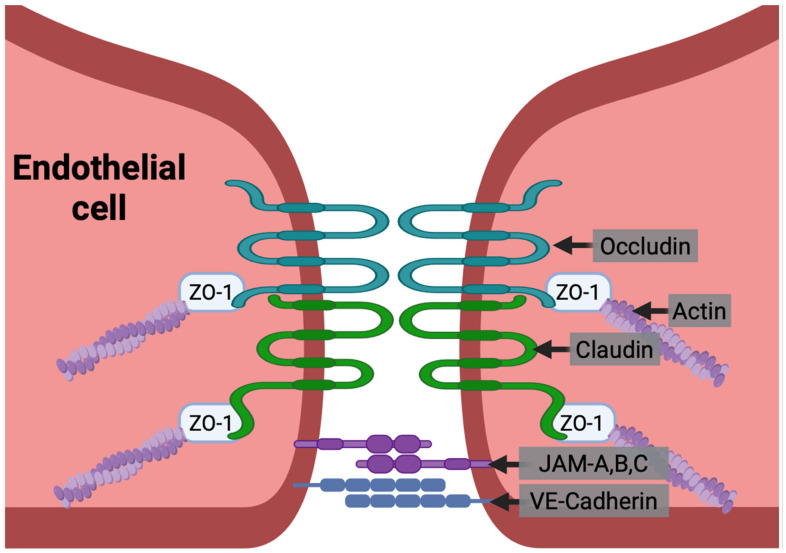
A diagram of tight junction proteins, junctional adhesion molecules, and adherens junction proteins between endothelial cells. BBB permeability is regulated by specialized junctional structures that restrict paracellular transport between brain microvascular endothelial cells. Tight junctions, composed of claudin-5, occludin, and ZO-1, together with junctional adhesion molecules (JAMs), seal the apical borders of BMVECs and limit the passage of ions, solutes, and immune cells. Zona occludens (ZO) proteins anchor these transmembrane complexes to the actin cytoskeleton. Basal to the tight junctions, vascular endothelial (VE)–cadherin forms adherens junctions linked to catenins and the cytoskeleton, providing additional mechanical stability and contributing to overall barrier integrity. Figure created with Biorender by authors.

BMVECs are connected by tight junctions, which are formed by interactions of cell surface and intracellular tight junction proteins including claudin-5, occludin, zona occludens (ZO)-1 and other junctional adhesion molecules (JAMs) (Figure 2). These tight junctions maintain BBB integrity and inhibit paracellular transit of ions, inflammatory mediators, and cells into the CNS [15,16]. The claudins and occludins are transmembrane proteins that form the tight junctions at the apical surface of BMVECs and are essential for the impermeability of the tight junctions. JAMs are also transmembrane proteins of the immunoglobulin superfamily that help to regulate tight junction permeability and leukocyte transmigration. Zona occludens proteins are cytoplasmic scaffolding proteins that, along with other cytoplasmic adaptors, connect the transmembrane complexes to the actin cytoskeleton [19,21]. Vascular endothelial (VE)–cadherins form adherens junctions between endothelial cells basal to the tight junctions (Figure 2). They also help maintain the integrity of the barrier and are connected to the actin cytoskeleton by catenins [19].

The basolateral surface of the endothelium sits on a basement membrane that not only anchors and connects the cells but also serves as another barrier for substances and cells to enter the CNS. The basement membrane is composed of molecules secreted by BMVEC and pericytes including type IV collagens, laminin, nidogen, heparin sulfate proteoglycans, and other glycoproteins. The interaction of BMVECs and basement membrane/extracellular matrix proteins facilitates signaling processes that control BBB permeability and function [16,19].

### 2.2. Pericytes

Pericytes are embedded within the vascular basement membrane, where they can form direct connections with endothelial cells. Their processes extend across the endothelium, regulating vessel diameter and contractility to maintain cerebral blood flow and vessel stability. They also contribute to angiogenesis, modulate immune cell infiltration, exhibit phagocytic activity to clear metabolic waste, and secrete extracellular matrix proteins that reinforce vascular integrity. Overall, pericytes are important for both the development and maintenance of the BBB [15,16,19].

### 2.3. Astrocytes

Astrocytes are the most numerous glial cells in the CNS and key to maintaining CNS homeostasis, including water and ionic concentrations, and BBB function. Polarized cellular processes extending from astrocytes contact both neurons and the cerebral vasculature, making astrocytes important in relaying neuronal signals that regulate blood flow and BBB permeability. Astrocyte endfeet are connected to the glia limitans perivascularis, which is the basement membrane of the BBB on the brain parenchymal side. These endfeet surround the entire length of the vascular endothelium and pericytes of cerebral capillaries, contributing to BBB integrity. Astrocytes also secrete factors that maintain the BBB and are implicated in both BBB disruption and repair [16,19,23].

### 2.4. Immune Cells

Brain macrophages and microglia are resident CNS parenchymal cells that serve important innate immune functions in the brain in part by phagocytosing cellular debris and pathogens. In addition to their phagocytic function, they contribute to neuronal development, antigen presentation, repair of injured brain tissue, and extracellular signaling [19,24,25,26,27].

There are many subtypes of brain macrophage and microglia including border-associated macrophages (i.e., perivascular macrophages, leptomeningeal macrophages, choroid plexus macrophages, dural macrophages) and disease-associated macrophages and microglia, which all can differ in localization, function, and self-renewal capability [24,25]. Brain macrophages can be replenished by self-renewal or by peripherally derived monocytes. Under normal conditions, peripheral monocytes typically only supplement choroid plexus macrophages and dural macrophages [24]. However, in pathological conditions, there is an increase in peripheral monocytes entering the brain parenchyma, where they differentiate into monocyte-derived tissue resident macrophages and contribute to pools of choroid plexus, dural, perivascular, and leptomeningeal macrophages [24,25].

Different stimuli can polarize microglia into inflammatory (M1) or anti-inflammatory (M2) phenotypes that have different effects on BBB permeability. M1 inflammatory microglia are associated with BBB dysfunction and permeability, while M2 anti-inflammatory cells protect the integrity of the BBB [28]. Single-cell and single-nucleus RNA-sequencing studies identified microglia with unique, disease-specific response states beyond canonical M1/M2 classification, underscoring the heterogenous and dynamic nature of microglia [29].

Entry of immune cells, including neutrophils, monocytes, and T cells, into the brain is limited under normal conditions. However, these cells can be activated in response to injury, infection, or in different disease states, leading to their production of inflammatory mediators and reactive oxygen species (ROS) and increased transmigration that may damage the barrier [19,30,31]. Further research is needed to understand the role of peripheral immune cells, brain macrophages, and microglia in BBB maintenance and dysfunction.

### 2.5. Neurons

Although neurons are not a direct component of the BBB, they have a crucial role within the neurovascular unit. Neuronal signaling coordinates with other neurovascular unit cells to support BBB development, regulate cerebral blood flow, and modulate transporter activity that governs nutrient uptake and metabolic waste clearance [32]. Neurons also secrete regulatory factors, such as glial cell line-derived neurotrophic factor (GDNF), which increase the expression of junctional proteins such as claudin-5 and VE-cadherin, thereby maintaining tight junction integrity and overall BBB stability [33].

## 3. Peripheral Inflammation, BBB Disruption, and Depression in People Without HIV and People with HIV

### 3.1. Overview of Findings in People Without HIV

Evidence supports a role for inflammation in the pathogenesis of depression. During infection, increased inflammation induces sickness behavior in animals, characterized by lethargy, loss of appetite, sleep disturbances, and decreased libido, symptoms also observed in depression [34,35,36,37,38]. Sickness behavior is considered an adaptive response that conserves energy to facilitate recovery from infection; however, it also reflects inflammatory processes that contribute to depressive behavior.

Many studies demonstrate chronic elevations of inflammatory mediators such as TNFα, IL-1β, IL-6, and C-reactive protein (CRP) in individuals with depression [7,39,40,41,42,43,44]. Serum CRP concentrations, as well as cerebrospinal fluid (CSF) levels of TNFα and IL-1β, correlate with depression severity [7,45,46,47,48]. Anhedonia severity, a core symptom of depression, is also positively associated with multiple cytokines [49,50]. Administration of cytokines induces depressive-like behaviors in both animal and human studies, and individuals with autoimmune or inflammatory conditions including multiple sclerosis (MS), arthritis, and lupus report higher rates of depression [34,51,52,53,54,55,56,57,58]. One study found that elevated serum IL-6, but not CRP, was associated with depression severity and chronicity in cross-sectional and longitudinal analyses [59]. Neuroimaging studies add to this literature suggesting relationships between blood cytokine levels and brain function underlying reward processes [60,61]. Despite conflicting findings of the efficacy of anti-inflammatory drugs in treating depressive symptoms in randomized control trials (RCTs), several meta-analyses of these RCTs reported that treatment with anti-inflammatory agents, including non-steroidal anti-inflammatory drugs and cytokine inhibitors, reduces depressive symptoms in individuals with major depressive disorder [62,63,64,65,66]. Collectively, these findings underscore the role of inflammation in depression.

Chronic inflammation may contribute to depression pathogenesis through its effects on the BBB. Peripheral cytokines including TNFα and IL-1β are associated with increased BBB permeability and are also linked to depression in animal models and humans [10,15,17,18,67]. Chronic social stress in mice induced depressive behaviors accompanied by increased CNS influx of IL-6 and IL-1β, likely mediated by reduced expression of the tight junction proteins claudin-5 and occludin and increased BBB permeability, as evidenced by increased gadolinium and albumin-conjugated dye accumulation in the brain compared to control mice [17]. Similarly, TNFα increased BBB permeability to sodium fluorescein and induced a prolonged depressive-like state in mice [18]. People with major depressive disorder were also found to have reduced claudin-5 in the nucleus accumbens and prefrontal cortex [17,68]. Additional cytokines and chemokines mediated through NF-κb and other inflammatory pathways also contribute to BBB permeability and depression in both animal and human studies [53,69,70,71,72]. Together, these findings implicate BBB disruption as a potential mechanism in depression pathogenesis.

### 3.2. HIV-Associated Chronic Inflammation May Contribute to Greater BBB Disruption and Depression Risk in PWH

HIV infection induces chronic inflammation that persists despite viral suppression [10,13,14]. This inflammation is characterized by elevated cytokines including TNFα, IL-1β, and IL-6, as well as increased frequencies of certain PBMC subtypes including peripheral blood monocytes expressing both CD14 (the lipopolysaccharide co-receptor) and CD16 (the FcγIII receptor) [73,74]. As described above, these cytokines are associated with BBB disruption. Additionally, transmigration of highly HIV-infected PBMCs was shown to increase BBB permeability in a human BBB coculture model, although this has not yet been examined in the context of depression or in virally suppressed individuals [75]. Both inflammatory cytokines and peripheral immune cells, including CD14^+^CD16^+^ monocytes and T helper 17 (Th17) cells, have been associated with depression [53,72,76,77,78,79,80,81,82]. We will review experimental evidence connecting BBB dysfunction with depression in more detail in later sections.

### 3.3. Other Pathways That May Mediate BBB Dysfunction and Depression in People Without and with HIV

The kynurenine pathway: Inflammatory mediators may also contribute to BBB dysfunction and depression through activation of different metabolic pathways. One such pathway is the kynurenine pathway. The kynurenine pathway metabolizes tryptophan into kynurenine via indoleamine 2,3-dioxygenase (IDO), generating neuroactive metabolites including quinolinic acid and kynurenic acid. Quinolinic acid, an NMDA receptor agonist, promotes excitotoxic neuronal injury, while kynurenic acid, an NMDA receptor antagonist, is neuroprotective [83]. Inflammation upregulates IDO activity, leading to increased production of kynurenine pathway metabolites [83,84,85,86,87]. These metabolites are associated with BBB disruption, increased infiltration of inflammatory monocytes into the brain, and depressive symptoms [87,88,89,90,91]. Activation of the kynurenine pathway has also been associated with depression and anhedonia across age groups, as well as with changes in reward neurocircuitry [92,93,94,95,96,97]. While some studies have not found differences in absolute concentrations of peripheral kynurenine pathway metabolites between those with and without depression or depressive symptoms, ratios of metabolites, indicating IDO activity or neuroprotection, have been found to be associated with depressive symptoms.

In people without HIV, several studies show that the relative distribution of kynurenine pathway metabolites, rather than absolute metabolite concentrations, relates to depression. Reduced peripheral kynurenic acid and lower neuroprotective indices (e.g., kynurenic acid/quinolinic acid (KA/QA), kynurenic acid/3-hydroxykynurenine (KA/3-HK)) have been reported in major depressive disorder compared with controls, and these indices are associated with reduced hippocampal volume [83,92,98,99]. Our group similarly found that lower kynurenic acid was associated with greater anhedonia severity in women without HIV [100]. Together, these findings suggest that reduced neuroprotection within the kynurenine pathway may contribute to depressive symptomatology and neural vulnerability in PWoH.

Findings in people with HIV further implicate kynurenine pathway activation in depression pathogenesis, although results are inconsistent. In a study of PWH from Uganda, higher kynurenine/tryptophan (KYN/TRP) ratios, a marker of IDO activity, were positively associated with depressive symptoms, and ART-mediated reductions in KYN/TRP partially explained improvements in depression [101]. In another cohort, however, KYN/TRP did not mediate the association between HIV and depressive symptoms measured by the PHQ-9 [102]. Another study reported that lower plasma KYN/TRP was associated with major depressive disorder in PWH, although this association did not remain significant after correction [103]. More recently, one report showed that PWH with depression had higher quinolinic acid and QA/KA ratios, and both quinolinic acid and QA/KA were independently associated with depression measured by the Major Depression Inventory [104]. Consistent with this, our group found in a cohort of women with HIV that higher quinolinic acid was associated with greater depression and anhedonia severity, while lower kynurenic acid was also linked to worse depressive symptoms [100].

These studies suggest that kynurenine pathway dysregulation, particularly shifts toward the neurotoxic branch, may contribute to BBB dysfunction, neuroinflammation, and reward circuit alterations relevant to depression, especially in PWH. However, differential findings across studies may reflect heterogeneity in sample size, sex composition, comorbidities, ART status, metabolites measured, and how depression is defined (diagnosis vs. symptom severity; active vs. remitted vs. recurrent depression). Future studies should include well-characterized populations, comprehensive panels of inflammatory mediators and kynurenine pathway metabolites, and harmonized assessments of depression and related symptoms to facilitate comparisons and clarify the role of kynurenine pathway activation in HIV-associated depression pathogenesis.

The microbiome and BBB dysfunction: The microbiota–gut–brain axis has been implicated in the pathogenesis of neurocognitive and neuropsychiatric disorders in both PWoH and PWH. The gut microbiome can also affect BBB integrity. PWH exhibit greater gut microbiome dysbiosis and impaired gut barrier function, with increased bacterial translocation and lipopolysaccharide (LPS) production, which may promote systemic and neuroinflammation. Alterations in the gut microbiome, together with heightened inflammation, may contribute to BBB disruption and the development of depression, and these effects may be especially pronounced in PWH. While this interaction is important, the role of the microbiome in BBB dysfunction and neuropsychiatric disorders is beyond the scope of this review and has been reviewed elsewhere [105,106].

In summary, inflammatory mediators and immune cells have independently been associated with BBB disruption and depression, in studies of individuals with and without HIV [7,10,13,82]. Additionally, PWH have higher kynurenine pathway activity and neurotoxic kynurenine pathway metabolites compared to PWoH [104,107,108,109], which may further contribute to BBB disruption and depression. However, few studies have directly examined the interplay among HIV, inflammation, and BBB dysfunction in depression pathogenesis. Given that HIV is a chronic inflammatory condition, PWH may have higher depression prevalence due to elevated inflammatory cytokines, activated immune cells, and neurotoxic kynurenine pathway metabolites that together promote BBB disruption and depressive pathophysiology.

Further research is needed to identify which mediators and cell populations are involved, and by what mechanisms they disrupt the BBB and contribute to depression in the context of HIV. Additionally, more RCTs and meta-analyses are needed to assess how anti-inflammatory drugs may affect depressive symptoms specifically in PWH.

Below, we describe current experimental and clinical approaches used to examine inflammation-associated BBB dysfunction and its association with depressive symptoms in both non-HIV and HIV contexts.

## 4. In Vitro and In Vivo Approaches to Study the BBB

Normal and pathological BBB function can be assessed using both in vitro and in vivo approaches. In vitro BBB models have been developed using BMVECs, pericytes, astrocytes, and microglia derived from various species, immortalized cell lines, induced pluripotent stem cells (iPSCs) [110], and primary human cells [111]. BBB integrity and function are commonly examined using transwell systems composed of endothelial cell monolayers, cocultures of endothelial cells with astrocytes or pericytes, or more complex tricultures incorporating multiple components of the neurovascular unit [111,112,113]. These cultures are often grown on transwell inserts that separate the culture well into peripheral (apical) and CNS (basolateral) compartments, enabling directional studies of transport and barrier permeability. Once established, BBB models can be used for a range of assays. These include permeability assays using fluorophore- or dye-labeled dextran or albumin substrates, transendothelial electrical resistance (TEER) measurements to assess barrier tightness, imaging-based assays to visualize tight junction and other BBB proteins, and leukocyte transmigration assays [111,114]. Our group established a human BBB model consisting of primary BMVEC and primary astrocytes cocultured on either side of a transwell membrane. This model has been used extensively to study BBB permeability and transmigration of uninfected/HIV-infected human cells [75,111,115,116,117,118,119,120]. Similar functional assessments have also been applied to in vivo animal studies, animal studies using ex vivo brain tissue, and post-mortem human tissue, providing a bridge between in vitro findings and the in vivo physiology of the BBB.

In addition to transwell systems, organoid and microfluidic models have recently emerged as alternatives that may more closely recapitulate the 3D structure and physiological dynamics of the BBB [121,122]. These models use organoids and/or microfluidic chambers with or without media flow. Microfluidic chambers with media flow emulate the shear stress of circulation. Most 3D organoid models differentiate human iPSCs into complex organoids containing three or more cell types, capturing the heterogeneity of the BBB environment [123,124,125]. These platforms also enable both imaging-based analyses and functional assays to measure permeability [124,125,126]. Several organoid models incorporated HIV infection, with some infecting iPSC-derived microglia separately from the organoid [127,128], while others expose the entire organoid to virus [129].

In vivo, BBB permeability can be assessed in both animals and humans using neuroimaging techniques such as positron emission tomography (PET), dynamic contrast-enhanced (DCE) MRI, and a novel water-extraction-with-phase-contrast-arterial-spin-tagging (WEPCAST) MRI technique, enabling analysis of in vivo regional and global BBB permeability [130,131,132,133]. These approaches provide an understanding of mechanisms underlying BBB disruption, including endothelial cell dysfunction, altered tight junction protein expression and organization, and glial cell activation or dysfunction.

## 5. Mechanisms of Inflammation-Associated BBB Disruption

### 5.1. Endothelial Cell Dysfunction

As endothelial cells constitute the initial barrier between the circulation and CNS, they regulate the passage of solutes and immune cells. Upon activation or injury, whether from circulating stimuli or CNS-derived signals, the BBB can become more permeable, allowing for increased entry of soluble factors and cellular infiltrates.

Effects of soluble mediators on endothelial cells and tight junctions: Numerous studies demonstrate that circulating cytokines, inflammatory molecules, and other soluble mediators activate endothelial cells and increase BBB permeability. Experiments with endothelial cell monocultures characterize specific effects of inflammatory mediators on the endothelium alone and their molecular mechanisms. Western blotting, qPCR, flow cytometry, and ELISA are used to quantify changes in gene and protein expression following exposure to inflammatory molecules and viral proteins [119,134]. Immunofluorescence assays further enable visualization of cell adhesion molecules and tight junction protein expression and localization [114,119,134,135].

Endothelial activation can be induced by a variety of stimuli, including cytokines such as TNFα and IL-1β, purinergic agonists, and other inflammatory mediators [136,137]. Activation leads to transcriptional changes, upregulation of selectins and adhesion molecules on the endothelial surface, cytoskeletal remodeling, and tight junction reorganization [138,139,140]. Decreased expression and relocalization of tight junction proteins play a major role in BBB disruption. TNFα, IL-6, IL-8, IL-1β, and other inflammatory mediators induce downregulation and redistribution of junctional proteins away from endothelial cell borders, leading to increased permeability [69,70,71,119,141,142,143]. For example, IL-8 stimulation of endothelial monolayers reduced occludin, claudin-5, and ZO-1 mRNA and protein levels by more than 50%, relative to untreated, with occludin relocalizing away from intercellular junctions [141]. In a coculture model using CD34^+^ stem cell-derived BMVEC and pericytes, TNFα and IL-1β exposure increased permeability two-fold relative to untreated and altered tight junction protein expression, characterized by elevated claudin-5 protein mislocalized to the cytoplasm and decreased occludin and ZO-1 at both the mRNA and protein levels [142]. Consistent with these findings, both in vitro and animal studies demonstrate that IL-1β and TNFα act directly on BMVEC to increase BBB permeability, in part through NF-κb, p38 MAPK, ERK1/2, and PI3K signaling pathways that regulate tight junction protein expression and localization, as reviewed elsewhere [144].

Additional mediators also contribute to BBB dysfunction. In one study using a BBB model of human astrocytes and a human immortalized endothelial cell line, treatment with a purinergic receptor agonist induced IL-1β and matrix metalloproteinase-9 secretion, as measured by ELISA. Western blotting showed that ZO-1 and occludin decreased by 60 and 30%, respectively, and the barriers were more permeable to FITC-dextran, relative to untreated. In other studies, LPS or growth factors TGF-β and VEGF exerted similar barrier-disrupting effects on endothelial cells [113,145,146,147]. The kynurenine pathway metabolite quinolinic acid is also implicated in BBB dysfunction. Quinolinic acid increased BBB permeability in rats, and one study found an association with quinolinic acid and BBB permeability in humans [87,88,89,90].

It is important to note that many studies using immunofluorescence imaging of tight junction proteins provide primarily qualitative descriptions of changes in expression and localization rather than quantitative measurements. Additionally, because baseline conditions and permeability assays vary across studies, comparisons are generally valid within a given experiment, but absolute values are not necessarily comparable between studies. Differences in treatment duration, mediator concentration, and the cell types used further limit direct cross-study comparisons. Despite these methodological differences, findings consistently demonstrate reduced tight junction protein expression and/or altered localization in response to inflammatory mediators.

In vivo and clinical studies further support these molecular findings. LPS treatment in mice is associated with increased peripheral inflammation and BBB disruption, as evidenced by increased FITC-dextran or Evans blue dye-coupled albumin uptake into brain parenchyma, up to two-fold compared to control, as measured by fluorescence [148,149,150]. Environmental stress models in mice demonstrate elevated peripheral inflammation, reduced BBB expression of claudin-5, and increased barrier permeability [17,68]. In MS-relevant experimental autoimmune encephalomyelitis (EAE) mice, induction of EAE was associated with ZO-1 disorganization at focal lesions of inflammatory cell infiltration and increased BBB permeability [151]. Post-mortem analyses from individuals with neurodegenerative and neuroinflammatory disorders including Alzheimer’s disease (AD), MS, amyotrophic lateral sclerosis, and HIV-associated dementia (HAD) further demonstrate endothelial degeneration and reduced or disrupted expression of claudin-5, occludin, and ZO-1 at the BBB [152,153,154,155,156]. Collectively, these studies underscore the central role of inflammatory mediators in promoting endothelial cell dysfunction and tight junction disorganization, which together contribute to BBB breakdown under pathological conditions.

Few studies directly examine inflammatory mediator effects on endothelial tight junction changes and BBB permeability in the context of virally suppressed HIV. However, many mediators described above and associated with BBB disruption in non-HIV contexts are also present in virally suppressed PWH and thus represent potential pathways in HIV-associated BBB dysfunction and depression pathogenesis. In addition, certain HIV proteins produced by HIV-infected cells can contribute to BBB disruption. These proteins continue to be produced despite effective ART [157]. Endothelial cells treated with Tat protein had decreased total ZO-1 expression and increased ZO-1 localization to the nucleus [158,159]. Additionally, treatment of endothelial cells with Tat or gp120 induced oxidative stress through the reduction in antioxidative machinery, resulting in endothelial cell damage [160]. Endothelial cells treated with gp120 also had disrupted tight junctions and downregulation of ZO-1, ZO-2, and occluding by 40–60%, depending on duration, relative to untreated cells as shown by Western blot [135]. Nef protein also reduced endothelial cell ZO-1 and disrupted BBB permeability in vitro [161]. Within the brain, HIV proteins can be taken up by uninfected bystander cells, which has been demonstrated to have deleterious effects to several cell types of the neurovascular unit [157]. Thus, studying how HIV proteins affect cells involved in the BBB is important to characterize how HIV infection induces BBB permeability. Future work focused on HIV-specific mechanisms of BBB disruption in PWH is warranted.

Effects of inflammatory cells on endothelial cells and tight junctions: Leukocyte transmigration is a tightly regulated, multistep process that occurs sequentially through rolling, firm adhesion and crawling, and diapedesis [162,163]. Under inflammatory conditions, several changes occur in the endothelium that promote leukocyte entry into the CNS. These include, but are not limited to, (1) upregulation of selectins and cell adhesion molecules on the endothelial surface that facilitate rolling, (2) increased expression of integrins on the endothelial surface that mediate firm adhesion and arrest, and (3) increased presentation of chemokines such as CCL2, which bind to their receptors on the leukocytes and enhance firm adhesion and arrest by activating integrins on these cells [164,165,166,167]. Together, these molecular changes increase recruitment and transmigration of peripheral immune cells into the brain parenchyma.

Leukocytes can migrate across the endothelium through either paracellular or transcellular routes [168]. During paracellular transmigration, endothelial cells undergo cytoskeletal remodeling and transient disassembly of tight junctions, enabling leukocytes to pass between adjacent cells [168,169,170]. This process is accompanied by increased production of matrix metalloproteinases (MMPs) by infiltrating immune cells, which degrade components of the vascular basement membrane to facilitate entry [171]. While these changes to tight junctions and basement membrane integrity are most often reversible, chronic or repeated inflammatory activation may result in lasting BBB impairment. Leukocytes can also undergo transcellular transmigration directly through endothelial cells, with a transcellular pore being initiated by leukocyte podosome extension [168,169,170,172]. Both methods of transmigration may have distinct molecular requirements involving specific molecules, but they also share overlapping mechanisms, including formation of a migratory cup and engagement of the endothelial lateral border recycling compartment [173,174,175,176]. Cell type, size, gene expression profile, inflammatory state, and which path offers least resistance may determine whether a cell undergoes paracellular or transcellular transmigration [175,177]. Repeated transmigration through either route may contribute to endothelial cell damage and increased BBB permeability. HIV infected T cells, and perhaps monocytes, can undergo either paracellular or transcellular transmigration in vitro but it is not known which method is preferable for HIV infected cells in vivo [178].

In inflammatory conditions such as HIV infection, increased leukocyte transmigration may contribute to sustained BBB disruption through endothelial damage, tight junction destabilization, and degradation of the basement membrane. This may result from repetitive junctional opening and resealing or from excessive production of inflammatory mediators, ROS, and MMPs. In vitro, transmigration of highly HIV-infected PBMCs was shown to increase BBB permeability in human coculture models, an effect not found with uninfected cells [75]. Among PBMC subsets, CD14^+^CD16^+^ monocytes, particularly those harboring HIV DNA, cross the BBB in greater numbers than other monocyte subsets and exhibit a more inflammatory phenotype, potentially exacerbating endothelial injury [118]. Recent work identified a subset of CD14^+^CD16^+^ monocytes that are more inflammatory and cross the barrier more efficiently compared to other CD14^+^CD16^+^ monocytes, suggesting they may cause greater damage to the BBB [179]. Similarly, Th17 cells, which are implicated in the pathogenesis of MS and likely depression, demonstrate a higher capacity for BBB transmigration than other T cell subsets and may contribute to barrier dysfunction through secretion of IL-17 and IL-22 [180].

Further research is needed to determine whether activated immune cells in chronic inflammatory conditions cause persistent structural or functional BBB damage, and to determine whether such injury arises from transmigration itself or from the secretion of BBB-disrupting factors, or both. Specifically, studies in the context of HIV should identify which immune cell populations are most responsible for BBB disruption and the mechanisms by which they impair barrier integrity.

### 5.2. Mural and Glial Cell Dysfunction

Other components of the structural and neurovascular unit, including astrocytes, pericytes, and microglia, regulate endothelial function and BBB integrity. Loss, alterations in cell morphology, and activation of these cells can all contribute to BBB disruption [152].

Both astrocytes and pericytes, as well their appropriate morphology, are needed to maintain BBB structure and function. Gliotoxin-mediated loss of astrocytes in a rodent model resulted in decreased paracellular localization of claudin-5, occludin, and ZO-1 and increased leakage of dextran and fibrinogen into the brain [181]. Pericyte loss has also been associated with increased BBB permeability in both mouse models and human studies [152,182,183].

Activated astrocytes and pericytes produce inflammatory cytokines and molecules that are implicated in tight junction reorganization, decreased tight junction proteins, and basement membrane degradation [144,184,185,186,187]. Under normal conditions, astrocyte-secreted factors like glial-derived neurotrophic factor, fibroblast growth factor, and angiopoietin-1 support BBB integrity and function [144,188,189,190,191]. In neuroinflammatory states, however, astrocytes become reactive and secrete VEGF-A, MMP-9, and CCL2, which can disrupt tight junctions, degrade the basement membrane, and enhance leukocyte transmigration across the barrier [184,185,186,187]. Astrocyte-derived IL-6 was specifically shown to increase permeability and alter tight junction architecture [192], while exposure to TNFα drove iPSC-derived astrocytes toward an inflammatory phenotype that impaired BBB integrity when cocultured with BMVEC through activation of the STAT3 pathway [193]. Similarly, inflammatory stimulation of pericytes promotes secretion of cytokines IL-6 and MIP-1α, which contribute to BBB dysfunction [194]. Mediators released by both astrocytes and pericytes can also activate microglia, amplifying cytokine, chemokine, and ROS production that exacerbate endothelial injury. This inflammatory environment promotes leukocyte transmigration across the barrier. Infiltrating immune cells can release more mediators to activate glial cells and disrupt the barrier, perpetuating a cycle of neuroinflammation and BBB disruption.

Experimental evidence supports the detrimental impact of activated glia on BBB function. In a coculture model of rat microvascular endothelial cells and microglia, LPS-activated microglia increased permeability of the barrier to sodium fluorescein, a dye which crosses the barrier through the paracellular route when the BBB is damaged [195]. Additionally, stimulating mixed glial cultures with LPS induced secretion of inflammatory cytokines, and coculture of the stimulated mixed glial cells with BMVEC resulted in increased barrier permeability and subcellular redistribution of tight junction proteins, demonstrating that activated glial cells directly impair BBB integrity [196]. Despite differences in the coculture models, durations, concentrations, and methods used, LPS increases BBB permeability, as measured by permeability to fluorescein or mannitol or by reduced resistance by TEER.

In the context of HIV infection, our group showed that astrocyte infection and activation disrupt gap junctions and increase BBB permeability [120]. HIV-infected astrocytes can also induce apoptosis of bystander, uninfected cells in vitro and were closely associated with apoptotic endothelial cells of the BBB in a monkey model [120,197].

Pericytes have the potential to become infected by HIV because they express CD4 and co-receptors CXCR4 and CCR5 [198,199,200]. In a human in vitro coculture model of BMVECs and pericytes, infecting pericytes with HIV disrupted BBB integrity [199]. Both astrocytes and pericytes have been shown to be infected in post-mortem tissue of PWH [157,200]. However, post-mortem studies assessing HIV-1 infection of pericytes were limited to PWH not on ART. Thus, no published data evaluate pericyte infection in virally suppressed PWH, and whether pericytes from treated individuals behave similarly to those in vitro and post-mortem studies in terms of infection and BBB disruption remains unknown [201].

Nonetheless, given that HIV activates multiple neurovascular unit cell types, these processes represent possible mechanisms of BBB disruption in PWH. Further studies are needed to delineate the specific mediators and cell types most responsible for BBB impairment in PWH, which may identify novel therapeutic targets to preserve barrier function in this population.

## 6. Studies That Address BBB Disruption and Depression

### 6.1. Animal Models of Depression

Animal models of depression provide valuable tools for examining the molecular and cellular mechanisms underlying depression pathogenesis as reviewed elsewhere [202]. Depressive-like behavior in these models is induced through paradigms such as chronic social defeat, chronic unpredictable mild stress, inescapable foot shocks, early-life stress (e.g., maternal separation), or glucocorticoid administration. Behavioral assessments used to evaluate depressive-like phenotypes include the forced swim and tail suspension tests, where decreased mobility reflects behavioral despair; learned helplessness paradigms, where reduced shock avoidance indicates hopelessness; and the elevated plus maze or open field test, where reduced exploratory behavior and locomotion signal anxiety-like symptoms. Anhedonia can be measured through decreased sucrose preference, while physiological alterations such as weight loss and elevated cortisol levels further parallel depressive symptomatology in humans [202].

These models consistently reproduce behavioral and physiological changes, including reduced social interaction, increased defensive behavior, disrupted sleep, immune dysregulation, and activation of the hypothalamic–pituitary–adrenal (HPA) axis [202]. After induction of depressive-like behavior, ex vivo brain tissue can be used to examine neural circuits and signaling mechanisms underlying these behavioral changes using in situ hybridization methods [203]. Additionally, animal models allow researchers to study, through pharmacological, genetic, and optogenetic manipulations, the mediators, BBB, and neuronal populations involved in depression-related behaviors in vivo, which are approaches not feasible in human studies [202,204].

### 6.2. Animal Studies of BBB and Depression

Multiple studies support a role for tight junction alterations and BBB disruption in depression pathogenesis. In one study in mice, chronic unpredictable mild stress induced depression-like behaviors including reduced sucrose preference and prolonged immobility in tail suspension and forced swim tests. These behavioral changes were accompanied by sodium fluorescein leakage into the brain and decreased gene and protein expression of claudin-5 and ZO-1 compared to control mice [205]. In other studies, chronic social defeat stress and chronic unpredictable stress resulted in decreased social interaction in stress-susceptible mice compared to resilient mice, which was associated with decreased gene expression of claudin-5 in the nucleus accumbens and pre-frontal cortex, key regions in reward neurocircuitry [17,68]. Additionally, male stress-susceptible mice and mice with conditional knock-out of claudin-5 displayed increased BBB permeability with increased entry of peripheral IL-6 and development of depression-like behaviors [17]. In female mice, conditional knockout of claudin-5 in the prefrontal cortex also resulted in anxiety and depression-like behaviors [68]. In a prolonged learned helplessness model, mice that failed to recover from induced learned helplessness demonstrated increased BBB permeability and elevated hippocampal levels of TNFα, IL-17A, and IL-23. Treatment with immune-modulating agents Fingolimod or TNFα inhibitors restored normal behavior, increased tight junction protein expression, and reduced hippocampal cytokines in these non-recovered mice [18].

There are few studies examining the intersection of BBB dysfunction and depression pathophysiology in mouse HIV models. One study using mice infected with EcoHIV, a chimeric virus that replicates in mouse CD4+ T cells, macrophages, and microglia and recapitulates HIV-associated neurocognitive impairment, showed that EcoHIV-infected mice expressed depressive-like behaviors including decreased social interaction, decreased sucrose preference, and sleep disturbances without using other paradigms to induce these behaviors. Treatment with a glutamine antagonist ameliorated these behaviors. However, BBB permeability was not assessed in this study [206]. Another study with the EcoHIV-infected mouse model found that these mice had decreased expression of claudin-5 and increased BBB permeability, as measured by sodium fluorescein intravasation into the brain, compared to control mice. However, this study did not assess depressive-like symptoms [207]. Further research is needed to characterize the relationship and mechanisms underlying HIV-associated BBB disruption and depression pathogenesis.

### 6.3. Limitations of Animal Studies

It is difficult to compare animal models because they use different paradigms to induce depressive-like states. While many studies assess several depressive phenotypes, no model can fully recapitulate all symptoms of human depression. In addition, species-specific differences in the BBB limit the generalizability of animal findings to humans, underscoring the importance of complementary clinical and post-mortem human studies. Although there is substantial heterogeneity in how stress or depressive phenotypes are induced, most models capture core behaviors including hopelessness, despair, anxiety, and fear. However, the lack of a standardized approach makes direct comparisons across studies challenging.

Despite these limitations, the range of available animal models enables investigators to examine discrete behaviors and symptoms relevant to depression. Consistently across models, decreased tight junction protein expression and/or altered localization in BMVEC, along with increased BBB permeability, are found in association with heightened systemic inflammation. Animal studies remain essential for testing mechanisms and performing genetic or pharmacologic manipulations that are not feasible in humans and are necessary to establish causality. Additional work using animal models is necessary because relatively few studies have examined the mechanisms linking BBB dysfunction and depression, particularly in the context of HIV.

### 6.4. Human Studies of BBB and Depression

A number of human studies have implicated BBB dysfunction in the pathogenesis of depression [17,68,208]. Post-mortem brain tissue from individuals with depression are important for identifying neuroanatomical, pharmacological, and biochemical mediators in the pathophysiology of BBB disruption and depression [209]. Additionally, in vivo neuroimaging including WEPCAST MRI and DCE-MRI provides measures of global and regional BBB permeability, respectively. These neuroimaging techniques have been used to detect in vivo BBB permeability in studies of people with major depressive disorder, AD, and other neurodegenerative/neuroinflammatory disorders [131,210,211,212,213]. The different imaging modalities used to measure BBB disruption are compared in Table 1.

Findings in PWoH: Post-mortem studies report decreases in claudin-5 in the nucleus accumbens and hippocampus of individuals with depression compared to healthy controls [17,224]. Recent studies using DCE-MRI showed that BBB permeability is higher in people with major depressive disorder compared to healthy controls and in individuals with bipolar disorder experiencing more severe depressive symptoms compared to those with less severe symptoms and to healthy controls [213,218].

Findings in PWH: As discussed below there are limited studies using neuroimaging in PWH. DCE-MRI has been used to assess BBB permeability in PWH with HIV-associated neurocognitive impairment (HIV-NCI), showing significantly increased regionally specific BBB permeability in PWH with HIV-NCI compared to those without HIV-NCI [217].

Limitations: Studies of BBB disruption in PWoH with depression remain limited, and there are currently no published studies examining BBB disruption in PWH and comorbid depression. Similarly, no longitudinal studies examining the BBB in PWoH and PWH with depression have been reported. Additional work is needed to determine whether existing findings are consistent across cohorts, to characterize potential sex differences in the relationship between BBB disruption and depression, and to understand how this relationship is affected by HIV and other comorbidities.

Several studies report sex differences in peripheral inflammatory cytokines associated with depressive symptoms [225], kynurenine pathway activity [226,227], inflammation-associated BBB damage [228], and BBB-related gene expression [228]. These differences in inflammatory mediators and BBB biology may contribute to the higher prevalence of depression in women compared with men. It also remains to be determined whether these differences persist or are amplified in PWH.

Although most studies linking inflammation, BBB dysfunction, and depression are correlative, they identify important associations that warrant further investigation using longitudinal human studies to assess temporal relationships, as well as in animal models to define causality, mechanisms, and potential therapeutic targets.

### 6.5. Markers of BBB Damage in People with and Without HIV

BBB damage can also be assessed through the presence of molecules in peripheral blood or CSF that are not usually present in these compartments.

Findings in PWoH: People with major depressive disorder have elevated CSF amyloid beta (Aβ) and an increased CSF-to-serum albumin ratio, indicating BBB disruption [229,230]. Elevated serum S100B, soluble E-selectin (sE-selectin), and neurofilament light chain (NfL) may also serve as markers of BBB damage. S100B is a marker of astrocyte damage, while NfL indicates neuronal damage [231,232]. S100B is not found in the peripheral circulation unless there is BBB disruption [231,233,234]. NfL is released into the blood and CSF as a result of neuronal injury [7,235,236]. BBB damage may further increase circulating NfL [235]. Recent studies found associations with elevated peripheral S100B and NfL and depression [231,232,236,237]. Soluble E-selectin may also be a marker of BBB disruption. E-selectin is increased on vascular endothelium during inflammation and shed into the circulation, forming sE-selectin. Although sE-selectin is a nonspecific marker of endothelial cell damage, it may serve as marker of BBB disruption, as it has been found to be elevated in the serum of women with major depressive disorder. In the same study, decreased claudin-5 mRNA was found in the nucleus accumbens in post-mortem tissue from women with major depressive disorder, suggesting BBB disruption that may contribute to peripheral levels of sE-selectin [68]. However, larger clinical studies are needed to confirm this association.

Findings in PWH: In PWH, BBB impairment is suggested from findings of an elevated ratio of CSF to serum albumin, which was correlated positively with CSF NfL and inversely with N-acetylaspartate, a marker for neuronal integrity and mitochondrial function [238]. Despite suppression of CSF and plasma HIV RNA following the initiation of ART by PWH, BBB permeability and NfL levels did not significantly improve over one year of ART treatment [238]. Similarly, among 38 neurologically asymptomatic, treatment naïve PWH, no significant reduction in CSF to serum albumin ratio was detected in the two years after these participants began ART [239], suggesting that BBB impairment may not be completely reversed despite ART. There are no studies that evaluate these markers or the other markers in conjunction with in vivo BBB disruption in PWH with depression. These are important analyses to be performed in the future.

## 7. Relationship of BBB Disruption with Depression Pathogenesis

### 7.1. Mechanisms of Inflammation-Associated Neuronal Dysfunction

**Figure 3:**
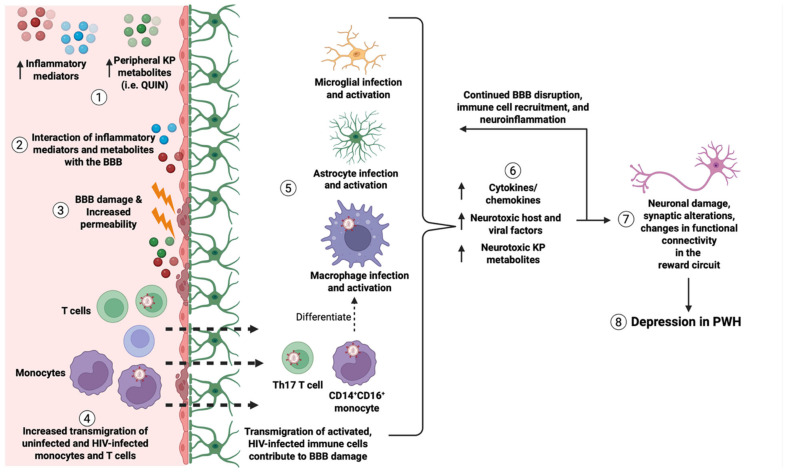
Proposed mechanism of inflammation-associated BBB disruption and depression pathogenesis in people with HIV. (1) Even with suppressive ART, HIV infection induces chronic inflammation with increased peripheral inflammatory mediators and kynurenine pathway metabolites (i.e., quinolinic acid). (2,3) BBB-disrupting peripheral inflammatory mediators and other metabolites interact with the BBB, causing BBB damage and increased permeability. (4) BBB disruption mediates increased infiltration of inflammatory factors and peripheral monocytes and T cells, particularly CD14^+^CD16^+^ monocytes and Th17 cells, into the CNS. Transmigration of activated HIV-infected immune cells may also contribute to BBB damage. (5) Within the CNS, activated and HIV-infected immune cells activate and infect resident CNS cells including macrophages, astrocytes, and microglia. Infiltrating monocytes also differentiate into tissue resident macrophages. (6,7) Activated and HIV-infected CNS cells and infiltrating immune cells produce cytokines/chemokines, neurotoxic host and viral factors, and neurotoxic kynurenine pathway metabolites that cause neuronal damage, synaptic alterations, and changes in functional connectivity in the reward circuit. These factors also recruit additional inflammatory cells into the CNS, perpetuating neuroinflammation. (8) Dysfunction of the reward circuit contributes to depression pathogenesis. KP, kynurenine pathway; QUIN, quinolinic acid; PWH, people with HIV. Figure created with Biorender by authors.

BBB damage caused by peripheral inflammatory mediators and activated transmigrating immune cells facilitates the entry of soluble factors and inflammatory cells into the CNS, leading to activation of resident macrophages and glial cells. Activated astrocytes, microglia, macrophages, and infiltrating immune cells release cytokines and chemokines that further recruit and activate immune and glial cells, perpetuating neuroinflammation. These mediators also contribute directly to neuronal injury and dysfunction (Figure 3). While there are not many published studies directly addressing the consequences of BBB disruption to neuronal damage in the context of depression, much can be learned from studies of neurodegenerative and neuroinflammatory disorders including AD and MS.

In AD, a neurodegenerative condition in which neuroinflammation is an important mediator, TNFα, IL-1β, and IL-6 can induce apoptosis, necroptosis, ferroptosis, and forms of mixed-modality cell death in neurons [240]. These same mediators also disrupt autophagy, alter synaptic structure, impair neuroplasticity, and activate signaling pathways including NLRP3 inflammasome, JAK–STAT, and RAGE/NF-κb [240,241,242,243,244,245]. Since the mediators that contribute to AD are also produced in the brains of PWH and people with depression, they may contribute to neuronal damage and therefore depression through similar mechanisms.

Inflammatory cytokines also regulate neurotransmission as reviewed elsewhere [246]. They alter the balance of excitatory and inhibitory neurotransmission, reduce synapse number and dendritic complexity, and disrupt neuroplasticity with effects that vary depending on cytokine composition, concentration, and cellular source [246]. In EAE mice, peripheral TNFα increased the turnover of dendritic spines and axonal boutons in the somatosensory cortex [247]. In another EAE study, neuron-derived TNFα increased cortical neuronal activity and produced anxiety-like behavior, both of which were reversed by treatment with a local TNFα inhibitor infliximab [248]. In AD-related models, type I IFN signaling drove microglia-mediated synapse loss and cognitive deficits, while blocking the IFNγ receptor or deleting the Type I IFN receptor in microglia reduced these outcomes [249,250]. Additionally, IL-17A–producing γδ17 T cells accumulate in the CNS of female AD transgenic mice and contribute to synaptic dysfunction and cognitive decline [251]. Thus, these factors, many of which are also associated with depression, may contribute to depression pathogenesis by altering synaptic function and neurotransmission.

In depression, inflammatory cytokines contribute to multiple pathophysiologic mechanisms, including HPA axis dysregulation, altered neurotrophic signaling (e.g., brain-derived neurotrophic factor, BDNF), changes in glutamate neurotransmission, and reduced neurogenesis [34,38,53,252,253]. Cytokines also disrupt neurotransmitter systems implicated in depression, including serotonin and dopamine [34]. Their induction of the kynurenine pathway further alters glutamate signaling through neuroactive metabolites. Quinolinic acid, an NMDAR agonist, increases glutamate release and impairs astrocytic glutamate uptake, contributing to neurotoxicity, while kynurenic acid, an NMDAR antagonist, reduces glutamate transmission and may counteract quinolinic acid-induced excitotoxicity [83,254,255]. Cytokines also reduce BDNF and increase phosphorylation of its receptor TrkB, altering receptor signaling and impairing neurogenesis and neuroplasticity [34,256].

In HIV, neurotoxic viral proteins contribute directly to neuronal injury [257,258]. PWH also express elevated quinolinic acid that contributes to neurotoxicity [104,107,108,257,259]. CD14^+^CD16^+^ monocytes, which are increased in the peripheral blood in PWH and in people with depression and implicated in HIV neuropathogenesis, may also contribute to HIV-associated depression, as they preferentially enter the CNS compared to other monocyte subsets and produce inflammatory mediators and ROS that can promote neuronal damage [82,179,258,260]. Th17 cells, which have been implicated in depression pathogenesis and associated with axon demyelination, may similarly contribute to HIV-associated depression [78,80,81,261,262,263]. For example, in a mouse model of intestinal inflammation, colitis led to increased BBB permeability, greater infiltration of Th17 cells into the brain, and depressive-like behaviors. Knockout of RORγt, the transcription factor required for Th17 differentiation, reduced T cell entry into the brain and prevented the development of depressive behavior [81]. The specific roles of both CD14^+^CD16^+^ monocytes and Th17 cells in HIV-associated depression remain to be characterized fully. Additionally, post-mortem tissue from PWH with HIV-NCI showed clusters of neurodegenerative glia and increased microglia/macrophage phagocytosis of neuronal synaptophysin, an essential synaptic vesicle protein for neurotransmitter release, compared to PWH without HIV-NCI [264]. These findings suggest that PWH exhibit mechanisms that disrupt neurotransmission and promote neuropathogenesis, which may also contribute to HIV-associated depression pathogenesis.

### 7.2. Selective Regional BBB Permeability and Depression Pathogenesis

Evidence suggests that BBB permeability exhibits regional specificity in neurodegenerative and neuroinflammatory diseases [265]. Such regional patterns of BBB permeability are likely disease-specific, and increased permeability in particular brain areas may result in region-targeted neuronal damage that contributes to distinct clinical manifestations. Functional MRI studies provide an understanding of brain regions involved in depressive behavior [266]. PET imaging can also be used to examine changes in brain metabolism, neuronal activity and connectivity, neurotransmitter interactions, and receptor binding in people with depression [267]. In depression, regions involved in reward processing appear especially susceptible (Figure 3).

The mesolimbic reward circuit involves projections of dopaminergic neurons from the midbrain ventral tegmental area (VTA) to the striatum, nucleus accumbens (NAc), prefrontal cortex, amygdala, hippocampus, and other limbic structures [268,269]. Structural and functional abnormalities have been reported across multiple components of this circuit in people with depression [266]. Studies demonstrate alterations in white and gray matter in the frontal lobe, hippocampus, temporal lobe, thalamus, striatum, and amygdala, along with disrupted functional connectivity among these regions [270,271]. Depression has also been characterized by reduced volume of the striatum, hypothalamus, and thalamus, and enlargement of the amygdala [272]. At the cellular level, decreased dendritic complexity, reduced synapse number, and dendritic atrophy have been observed in the prefrontal cortex and hippocampus [272].

Functional network alterations further highlight the vulnerability of these circuits. Hyperactivity of the default mode network (DMN), a network typically more active at rest, has been associated with increased self-focus, diminished attentional control, and persistent negative rumination [272,273,274,275]. The reward circuit includes regions belonging to the DMN, salience network, and limbic network [276,277]. Dysfunction within this circuitry is associated with anhedonia, a core symptom of depression that predicts greater depression severity, longer episode duration, and increased suicidality [278,279,280,281]. Our group and others demonstrated that alterations in reward neurocircuitry correlate with depression and anhedonia severity [282,283]. Studies in mice and human post-mortem tissue show that the nucleus accumbens and other reward-related structures exhibit downregulation of tight junction proteins, abnormal BBB morphology, and increased permeability [17,68].

Peripheral inflammatory challenges also affect reward circuitry, and structural and functional MRI studies have found associations between peripheral inflammatory mediators and regional changes in gray and white matter, atypical activation patterns, and changes in functional connectivity in depression [266]. Induction of peripheral inflammation by LPS administration or typhoid vaccination were associated with changes in basal ganglia activity, including reduced ventral striatal activation during reward processing, which correlated with greater depressive symptoms, and increased nigral activity associated with psychomotor slowing [61,284,285]. Additionally, people with depression showed decreased ventral striatal activation to positive stimuli compared to healthy controls, further suggesting reward circuit deficits in people with depression [286]. PWH also exhibited decreased ventral striatal activation during reward cue processing [287]. These findings suggest that disrupted connectivity or neuronal injury within reward-processing circuits may contribute to depression pathogenesis in PWoH and PWH. Increased BBB permeability within reward-related brain regions may mediate these abnormalities, with more BBB disruption allowing for greater entry of inflammatory mediators and leukocytes, which contribute to macrophage and glial activation and promote region-specific neuronal damage that contributes to depression (Figure 3). Mouse models of depression and post-mortem tissue from people with depression consistently show decreased tight junction proteins and increased BBB permeability in key reward circuit regions including the nucleus accumbens, dorsal striatum, hippocampus, and prefrontal cortex [17,18,68,263,288]. In HIV, these same regions may exhibit increased permeability, although research on this is limited. Additional work is necessary to identify factors that confer susceptibility or resilience to regional BBB permeability and to determine whether regional BBB disruption precedes disease onset or is a consequence of ongoing pathology.

### 7.3. Damage of Specific Neuronal Populations and Depression Pathogenesis

Neurons in the reward circuit, specifically dopaminergic neurons, may be more susceptible to neuronal injury. This may be due to their high energy demand, which makes them particularly sensitive to metabolic disturbances including mitochondrial dysfunction and oxidative stress [289,290]. Inflammation can contribute to these metabolic disturbances. Brain regions rich in dopaminergic neurons such as the ventral midbrain are also more vascular due to their high metabolic demand, and thus might be exposed to more peripheral inflammation, resulting in more disruption, CNS infiltration, and neuroinflammation to damage the neurons [265]. Studies have shown that striatal and cortical neurons are especially susceptible to neurotoxic kynurenine pathway metabolites, and quinolinic acid directly affects the mesolimbic dopaminergic system, inducing dopaminergic and GABAergic neuronal death [291,292,293,294,295].

As reviewed elsewhere [296], studies using an HIV transgenic rodent model, which expresses viral proteins but does not produce infectious virus and develops cognitive impairment similar to virally suppressed PWH on ART, demonstrate reduced dendritic spine density and altered neuronal transmission in the prefrontal cortex, hippocampus, and striatum. These studies show multiple forms of synaptic dysfunction, including hyperexcitability of pyramidal neurons in the PFC [297], decreased excitability of CA1 pyramidal neurons in the hippocampus [297], and dysregulated firing of dopamine D2 medium spiny neurons in the striatum [296,298]. Consistent with these findings, neuropathology studies of post-mortem tissue from PWH with HIV-NCI report synaptodendritic damage in the prefrontal cortex, hippocampus, and striatum [299]. Together, these data indicate that HIV is associated with neuronal injury in brain regions that are also implicated in depression. However, most published studies in PWH with HIV-NCI were conducted pre-ART or in individuals with AIDS, and neuronal changes in virally suppressed PWH on ART may differ. In addition, these studies primarily address HIV-related neuronal damage in HIV-NCI. To our knowledge, no studies have validated HIV-related neuronal damage specific to depression. Overall, more research is needed to examine the interplay of peripheral inflammation, BBB disruption, neuronal damage, and depression specifically in virally suppressed PWH. Future work should also examine whether specific neurons are implicated in HIV-associated depression pathogenesis.

## 8. Gaps in Knowledge and Future Directions

Both animal and human studies demonstrate independent relationships among peripheral inflammation, BBB disruption, and depression. However, few studies examine these associations in the context of HIV. Key questions remain regarding whether BBB damage contributes to depression pathogenesis in PWH and which inflammatory mediators and cell types are involved. Future research should (1) examine the associations of BBB disruption and depression in animal models and human studies, both cross-sectional and longitudinal; (2) identify inflammatory mediators, glial cells, and immune cells implicated in BBB disruption and depression pathogenesis, including the mechanisms by which they contribute to neuronal injury; and (3) identify serologic markers of BBB damage and depression, all in the context of HIV. Characterizing the processes underlying inflammation, BBB disruption, and HIV-associated depression pathogenesis may identify biomarkers of depression risk or severity in PWH and lead to development of more targeted therapies for treating or preventing depression in this population.

### 8.1. Gaps in Knowledge and Future Directions for Mouse Models of HIV and Depression

As described above, mice infected with EcoHIV exhibit depressive-like behaviors in the absence of other paradigms used to induce depression [206]. However, it is not known whether EcoHIV-infected mice are more susceptible to induced depressive behavior and how such induction would impact inflammation, BBB integrity, and peripheral immune cell transmigration. Combining mouse models of HIV and of depression would enable researchers to study these parameters systematically. In vivo BBB permeability could be assessed in these animals using two-photon microscopy or other techniques such as sodium fluorescein or FITC-dextran extravasation into the brain [222,300,301]. These studies could also be done in the context of acute HIV infection or viral suppression [302,303]. Flow cytometry or immunofluorescent staining could be used to identify cells that transmigrate into the brain of EcoHIV-infected mice after induction of depressive behavior and clarify their contribution to neuropsychiatric comorbidity. Genetic or pharmacologic manipulations could also be used to characterize mediators or immune cells that contribute to depressive behavior in EcoHIV and other mouse models of HIV infection. For example, knockout or pharmacologic inhibition of enzymes of the kynurenine pathway such as IDO would highlight the role of kynurenine pathway metabolites in depression pathogenesis [83,304]. A T cell-only HIV mouse model could be used to characterize the contribution of T cells to depression pathogenesis, while Th17 knockout HIV-infected mice could define the specific role of Th17 cells in depression pathogenesis [81,305]. Other mouse models, including myeloid only mice, would enable assessment of myeloid cell contributions to depressive-like behavior in HIV infection [306]. Treatment with cytokine-specific antibodies or inhibitors (e.g., TNFα, IL-1β, or IL-6 blockade) could determine whether inhibiting these mediators prevents or alleviates depressive symptoms in HIV-infected mice. Examining brain slices from depressed mice with HIV can be used to assess peripheral mediator and immune cell brain infiltration, glial activation, tight junction protein alterations, and neuronal injury. These studies may also identify brain regions with greater permeability, neuronal populations most vulnerable to injury, and specific mechanisms of neuronal damage or death. Neurons isolated from depressed mice with HIV could be analyzed using scRNA-sequencing to determine changes in gene expression in the context of HIV and comorbid depression.

### 8.2. Gaps in Knowledge and Future Directions for Human Studies of HIV and Depression

Previous studies show BBB disruption in several neurodegenerative and neuroinflammatory disorders [152]. However, few studies examine BBB dysfunction in people with depression, and none assess BBB disruption in PWH with comorbid depression [213,217,218]. Future studies should use in vivo BBB imaging techniques, such as WEPCAST or DCE-MRI, to evaluate global and regional BBB permeability in PWH with depression. Additionally, research is needed to identify circulating markers of BBB damage and depression. S100B and NfL are promising candidates given their associations with astrocyte and neuronal damage, respectively, and with depression [231,232]. These markers may serve as biomarkers that could be used for risk stratification or monitoring disease progression or severity.

Several approaches could be used to determine what immune cells infiltrate the CNS and are enriched with depression comorbidity. Our group is characterizing monocyte and T cell transmigration using PBMC from PWH with and without depression in an in vitro human BBB model. CSF samples from PWH with and without depression could also be examined, similar to other studies that profiled CSF immune cells in PWH [307]. Immunofluorescent staining of post-mortem tissue from PWH with depression could identify infiltrating immune cells and assess tight junction proteins, regional BBB permeability, and neuronal damage, paralleling proposed animal studies. Spatial transcriptomics of post-mortem brain tissue from PWH with depression would enable detailed assessment of cellular interactions among BBB cells, glia, macrophages, infiltrating immune cells, and neurons, and characterize the immune microenvironment that may contribute to BBB disruption, neuronal damage, and depression pathogenesis [308,309]. This method could also identify cells expressing viral transcripts, associating CNS viral reservoirs and localized BBB impairment.

Additional work should integrate analyses of peripheral inflammation, PBMC transmigration, brain spatial transcriptomics, in vivo BBB imaging, fMRI, and depressive symptom severity to characterize more completely the inflammatory and neurobiological mechanisms underlying depression in PWH. These studies may identify specific cell populations or inflammatory mediators that could be targeted to prevent or treat depression in PWH.

## 9. Conclusions

Converging lines of evidence suggest that chronic peripheral inflammation and neuroinflammation, BBB disruption, and neurocircuit-level changes interact to mediate depression pathogenesis, and that these processes may be especially relevant in PWH. HIV-associated inflammation, which persists even with effective ART, exposes the BBB to inflammatory mediators, toxic metabolites, and activated immune cells that may disrupt endothelial tight junctions, activate resident glial cells, and facilitate infiltration of inflammatory cells. CD14^+^CD16^+^ monocytes and Th17 cells may be particularly important in this process. These events can promote neuroinflammation, alter neurotransmission, and contribute to neuronal injury in brain regions that are structurally and functionally vulnerable, particularly within the mesolimbic reward circuit, contributing to depression pathogenesis. Animal and human studies independently support associations between inflammation, BBB dysfunction, and depression, yet the integrated contribution of these mechanisms to HIV-associated depression, especially in virally suppressed PWH, remains poorly defined.

Future work will require multimodal approaches that bridge mechanistic studies and clinical research, including HIV/depression mouse models, transmigration studies using PBMC from PWH and in vitro human BBB models, and human studies combining in vivo BBB imaging, fMRI of reward circuitry, CSF and blood biomarkers, and post-mortem spatial analyses of BBB and neuronal integrity. By identifying the key inflammatory mediators, cell populations, brain regions, and neuronal subtypes that drive BBB disruption and depression in PWH, these studies may indicate biomarkers for risk stratification. They may also suggest novel, targeted strategies to preserve BBB function, protect vulnerable neural circuits, and ultimately prevent or treat depression in this highly affected population.

## Figures and Tables

**Table 1:** Imaging modalities used to assess BBB permeability. These imaging techniques are discussed extensively in these reviews [214,215].

Imaging Modality	How BBB Permeability Is Measured	Resolution	Strengths	Limitations
**Dynamic Contrast-Enhanced MRI (DCE-MRI)**	Measures leakage of intravenously injected gadolinium-based contrast agent (GBCA) across the BBB into the brain. Increased leakage results in greater T1-weighted signal intensity. Quantifies volume transfer constant, *K*^trans^, the rate of contrast movement from plasma to extravascular space. Higher *K*^trans^ indicates greater vascular permeability [214,216].	High spatial and temporal resolution Enables region-specific measurement of *K*^trans^/BBB permeability.	Provides regional BBB permeability measures Applicable in both animal models and humans. Detected increased BBB permeability in neuroinflammatory and neuropsychiatric disorders and in PWH with HIV-NCI [131,211,212,213,217,218].	Requires intravenous contrast, which is relatively toxic and contraindicated in renal disease, contrast allergy, and pregnancy. GBCA is large and may be less sensitive to subtle BBB disruption, producing low-magnitude signal changes and reduced signal-to-noise ratio [214,215]. Lack of standardized protocols and scanner variability complicate cross-study comparisons [219]. Reduced cerebral blood flow can affect *K*^trans^ quantification [220].
**Water extraction with phase-contrast arterial spin tagging (WEPCAST)**	Uses arterial spin labeling (ASL) to magnetically label arterial blood water as an endogenous tracer. After labeling, a fraction of water enters tissue while non-extracted water remains in venous blood. ASL signal measured in the super sagittal sinus is used to calculate global water extraction fraction (E). Cerebral blood flow and E are used to derive BBB permeability-surface area product (PS), a global measure of BBB permeability [132].	Lower spatial resolution. Provides a global measure of BBB permeability.	Non-invasive with short acquisition time. Applicable in both animal models and humans. Water, a smaller molecule than GBCA, may better detect subtle changes in BBB permeability. Good test–retest reproducibility [132,221]. Detected increased BBB permeability in several neurodegenerative diseases, including AD [215].	Water enters the brain through multiple pathways (e.g., aquaporin-4), limiting specificity for BBB disruption [214]. Does not provide regional BBB measures. No published studies to date in depression.
**Positron Emission Tomography (PET)**	Measures radioligand decay to quantify tracer transport across the BBB. Radioligands can target specific BBB molecules and transporters. Glucose analog fluoro-2-deoxyglucose (FDG) is transported across the BBB by GLUT1; reduced brain FDG uptake may reflect decreased GLUT1 function and BBB dysfunction [215]. Quantifies tracer extraction fraction and BBB permeability–surface area product (PS) [130,215].	Provides measures of BBB transporter function and permeability.	High sensitivity [214]. Assesses both cellular metabolism and BBB function. Uses radiotracers targeting BBB transporters to evaluate specific protein function and its relation to permeability. Applicable in both animal models and humans.	Often requires separate scans to measure tracer transport rates and cerebral blood flow. New methods aim to address this limitation but require further validation [130]. Sensitive to changes in cerebral blood flow.
**Intravital microscopy**	Used to measure leakage of fluorescently labeled dextrans (FITC or TRITC) across the BBB into the brain. Often performed using two-photon microscopy [214,222].	High spatial resolution. Provides regional BBB permeability.	Provides understanding of cellular and molecular mechanisms of BBB disruption. Enables quantification and localization of BBB components, including tight junction proteins [223].	Limited to animal studies.

## Data Availability

No new data were created or analyzed in this study.
